# Expedient Treatment of a Collodion Baby

**DOI:** 10.1155/2011/803782

**Published:** 2011-10-15

**Authors:** Michael Chung, Jaime Pittenger, Stuart Tobin, Andrew Chung, Nirmala Desai

**Affiliations:** ^1^University of Kentucky College of Medicine, Lexington, KY 40506-9983, USA; ^2^Department of Pediatrics, University of Kentucky, Lexington, KY 40536-0284, USA; ^3^Department of Surgery, University of Kentucky, Lexington, KY 40536-0284, USA; ^4^David Geffen School of Medicine, University of California, Los Angeles, CA 90095, USA; ^5^Department of Neonatology, University of Kentucky College of Medicine, Lexington, KY 40504, USA

## Abstract

Only *~*270 cases of collodion babies have been reported in the literature since 1892. As the name suggests, the term “collodion baby” refers to a phenotype that can be characterized by a yellow, shiny, tight parchment-like membrane stretched over the skin. Although the collodion membrane is only an evanescent condition of the newborn, neonatal complications can occur in 45% of all collodion babies, leading to a mortality rate of *~*11% in the first few weeks of life. Most children born as collodion babies will spontaneously desquamate within 2 weeks, but may be as long as 3 months. Eventually, these children develop signs of one of several types of ichthyosis, which gives the skin the appearance of “fish scales.” We report a unique case of a Caucasian male that was born as a Collodion baby at the University of Kentucky Children's Hospital in Lexington, Kentucky. Although the impairment of the skin barrier function put the patient at risk for a number of complications, he improved significantly after being treated with emollients and antibiotics. In contrast to previous findings, we found that skin emollients were beneficial and did not increase the risk of infection.

## 1. Introduction

Only ~270 cases of collodion babies have been reported in the literature since 1892, when the term was first introduced by Hallopeau and Watelet [1, 2]. Collodion baby is a prodromal stage that precedes the true underlying disease entity. As the name suggests, the term “collodion baby” refers to a phenotype that can be characterized by a yellow, shiny, tight parchment-like membrane stretched over the skin. Observers may sometimes use the descriptor “dipped in hot wax.” [2] Although the collodion membrane is only an evanescent condition of the newborn, neonatal complications can occur in 45% of all collodion babies, leading to a mortality rate of ~11% in the first few weeks of life [3, 4]. Most children born as collodion babies will spontaneously desquamate within 2 weeks, but may be as long as 3 months. Eventually, these children develop signs of one of several types of ichthyosis, which gives the skin the appearance of “fish scales” [5]. 

We report a unique case of a Caucasian male that was born as a Collodion baby at the University of Kentucky Children's Hospital in Lexington, Kentucky. Although the impairment of the skin barrier function put the patient at risk for a number of complications, he improved significantly after being treated with emollients and antibiotics. In contrast to the findings of van Gysel et al., we found that skin emollients were beneficial and did not increase the risk of infection.

## 2. Case Report

We present the case of a Caucasian male infant who was born at 37 weeks' gestation to a 19-year-old, gravida 3, para 1001, mother. The mother received regular prenatal care, including prenatal vitamins and iron during pregnancy and also received Stadol for pain relief during labor. Toward the end of her pregnancy, the mother developed idiopathic thrombocytopenic purpura (ITP). Mother was hepatitis B negative, HIV negative, *Group B Streptococcus* negative, but rubella and VDRL were unknown. The patient was born by vaginal delivery with spontaneous rupture of membranes approximately 2 hours prior to delivery. Apgars were 9 and 9 at 1 and 5 minutes, respectively. He was given Vitamin K and Erythromycin eye ointment in the delivery room. 

The patient's birth weight was 2499 grams. Upon initial physical examination, the patient presented with a thin collodion membrane that was found over the majority of his skin surface. He was active and alert and appropriately responsive during the examination. The physical examination was unremarkable except for apparent inability to close the eyes completely and limited range of motion in all joints due to the taut collodion membrane. The patient's hearing was assessed to be normal, and his nails were normal in appearance and did not show any dystrophic changes, excluding the possibility of the keratitis, ichthyosis, and deafness (KID) syndrome. The patient could not purse his lips and was also noticed to have blanching of the eyelids. On day 7, he was noted to have slightly increased work of breathing due to upper airway obstruction from dried skin plugs. 

The patient was admitted to the neonatal intensive care unit (NICU) and placed in a humidified incubator with a cardiorespiratory monitor. At 6 hours of age, electrolytes were all within normal limits. Nasogastric feedings were initiated because of the patient's inability to suck. During his stay, the patient began daily bathing with either basis sensitive skin soap. Eucerin emollient cream and Aquaphor were also applied routinely every 3 to 4 hours. Once he began to crack and peel, Bacitracin zinc ointment was applied twice daily to the fissures in his skin to prevent infections. Lacri-Lube for the eyes was used every 4 hours to keep the corneas moist, and ammonium lactate was added as a keratolytic to augment the peeling process once the peeling started. He was placed on nasogastric feedings of NeoSure 22 calorie formula to increase protein intake for possible protein losses through the impaired skin barrier. Toward the beginning of his hospital stay, an extensive pedigree of the family was done, and his family history was unremarkable. Therefore, we concluded that this patient would, in all likelihood, demonstrate a self-healing collodion phenotype. 

During the 2nd week, the patient had diffuse peeling, especially in the facial area. Peeling around the eyes had also occurred, thus allowing him to close his eyes completely at this time. However, due to the persistent remnants of collodion membrane on his forehead, ammonium lactate treatment was continued so that his skin would completely exfoliate. On Day 27, the patient appeared pink, and his skin appeared to be much improved, but he still had significant edema of the lower extremities. On Day 29, the patient was started on a therapeutic amount of iron because he was found to have anemia with a hematocrit of 22.8% and absolute reticulocyte count of 76 k/*μ*L, most likely secondary to phlebotomy loss. On Day 31, the patient was then discharged from the hospital after stabilization of vital signs. 

During his hospital stay, a 7-day course of Nafcillin and Gentamicin were given for abdominal wall cellulitis. He also received Vancomycin for suspected coagulase negative Staphylococcus (CONS) sepsis, but this was subsequently discontinued as it was thought to be contaminant.

## 3. Discussion

We present the case of a male infant born as a Collodion baby, who after being placed in a humidified incubator and receiving expedient treatment with emollients and a prophylactic course of antibiotics, had a relatively uncomplicated hospital course. When the patient was 3 months of age, a 4 mm punch biopsy specimen was analyzed by a dermatopathologist. The presence of the granular layer and absence of dyskeratosis or acantholysis argued against ichthyosis vulgaris and epidermolytic hyperkeratosis. While lamellar ichthyosis and X-linked ichthyosis could have caused these changes, the working diagnosis was thought to be self-healing collodion baby since the patient's family history was unremarkable. X-linked ichthyosis is a clinical manifestation of steroid sulfatase deficiency, an inborn error of metabolism. Although this diagnosis can be elucidated by examining hairs by light microscopy, we did not feel it was necessary to perform an immunohistochemical analysis.

### 3.1. Other Forms of Ichthyoses

Ichthyoses represent a heterogeneous group of skin disorders that are characterized by a generalized scaling of the skin due to defective cornification and desquamation. Children born with autosomal recessive congenital ichthyosis (ARCI) often present at birth as collodion babies. In 1985, Williams and Elias classified ARCI into two major types using clinical, histological, biochemical, and molecular genetic criteria: nonbullous congenital ichthyosiform erythroderma (NBCIE) and lamellar ichthyosis (LI) [6, 7]. The third type of autosomal recessive congenital ichthyosis, Harlequin ichthyosis, is the most rare and severe form. The findings of van Gysel et al. corresponded closely with those of Larregue et al. with the most common outcome of collodion babies being erythrodermic autosomal recessive lamellar ichthyosis in 7 of 17 children (43% of cases), followed by nonerythrodermic autosomal recessive lamellar ichthyosis in 3 of 17 children (19% of cases), and other forms in 2 of 17 children (12% of cases). In 4 of 17 children (10% of cases), they found that the skin eventually developed normally, therein giving rise to a self-healing collodion phenotype, as is the case with our patient [1, 8].

One important distinction between autosomal recessive congenital ichthyoses and other forms of ichthyoses is the age of onset of symptoms. Whereas the three types of autosomal recessive congenital ichthyoses typically present with collodion membrane or ichthyosiform erythroderma at birth, other forms of ichthyoses such as ichthyosis vulgaris and X-linked recessive ichthyosis manifest after birth. In some cases, extracutaneous symptoms could be diagnostic clues for recognizing special syndromes with associated ichthyoses including the following: Sjogren-Larsson syndrome, Netherton syndrome, Ichthyosis prematurity syndrome, Gaucher syndrome type 2, Keratitis-ichthyosis-deafness (KID) syndrome, Trichothiodystrophy, Dorfman-Chanarin syndrome, congenital hemidysplasia with ichthyosiform nevus and limb defects (CHILD) syndrome, ichthyosis follicularis, atrichia, and photophobia (IFAP) syndrome, and Conradi-Hünermann-Happle syndrome [9]. Accordingly, these syndromic ichthyoses should be included in the differential diagnosis when a child presents with generalized scaling of the skin.

### 3.2. Neonatal Complications and Management of Collodion Babies

Due to mechanical compression, the parchment-like membrane in collodion babies may distort the features of the face and the extremities, giving the newborn a striking appearance that may initially frighten family members and physicians who may have never diagnosed such a case. The ears can appear malformed, the eyelids can be everted (ectropion), and the lips can be everted, giving a “fish mouth” appearance (eclabium) [6]. Due to the impairment of the skin barrier function, collodion babies are at risk for a number of complications, including hypernatremic dehydration, hypothermia, skin infections, fissures, conjunctivitis, sepsis, dehydration, and constrictive bands of the extremities resulting in vascular compromise and edema [1, 5]. The edema in the patient described here was thought to be due to either hypoproteinemia or mechanical compression by the collodion membrane. One study showed that the transepidermal water loss in collodion babies can be six to seven times higher than that through normal skin [10]. Therefore, it is essential that collodion babies be placed in a humidified incubator soon after birth to prevent hypernatremic dehydration and hypothermia. The patient described here was managed accordingly with the use of emollients and prophylactic antibiotics. In contrast to the findings of van Gysel et al., we found that the skin emollients facilitated skin healing and led to a relatively uncomplicated hospital course. 

## 4. Conclusion

Unfortunately, none of these clinical features in a collodion baby can be used to predict the final diagnosis or prognosis of the underlying ichthyosis phenotype. In addition, since the diagnosis of collodion baby is a clinical one, examining histopathologic features of skin biopsy specimens in the first few weeks will not be useful in differentiating the different types of ichthyosis [1]. Thus, in order to determine the etiologic cause for the collodion membrane, a protocol must be established so that appropriate measures can be taken months or years following the shedding of the collodion membrane, including the following: a good family history, a good clinical examination, histopathologic examination of skin biopsy specimens, and appropriate laboratory tests. It is important to note though that accurately diagnosing a collodion baby can be a difficult and long process, especially since patients may change from predominantly scaly skin to predominantly erythematous skin as they get older in age.
